# Integrating Genomic and Clinical Data in AML: Real-World Application of the Sanger Multistage Model

**DOI:** 10.3390/genes17020218

**Published:** 2026-02-10

**Authors:** Andrea Duminuco, Silvia Rita Vitale, Antonella Nardo, Patrick Harrington, Stefania Stella, Michele Massimino, Cristina Tomarchio, Elisa Mauro, Marina S. Parisi, Cinzia Maugeri, Francesco Di Raimondo, Giuseppe A. Palumbo, Livia Manzella, Calogero Vetro

**Affiliations:** 1 Hematology with BMT Unit, A.O.U. Policlinico “G. Rodolico-S. Marco”, 95123 Catania, Italy; antonella.nardo5@gmail.com (A.N.); marinaparisi@hotmail.it (M.S.P.); francesco.diraimondo@unict.it (F.D.R.); palumbo.ga@gmail.com (G.A.P.); 2 Center of Experimental Oncology and Hematology, A.O.U. Policlinico “G. Rodolico-S. Marco”, 95123 Catania, Italy; silviarita.vitale@gmail.com (S.R.V.); stefania.stella@unict.it (S.S.); michedot@yahoo.it (M.M.); cristina.tomarchio@hotmail.it (C.T.); manzella@unict.it (L.M.); 3 Department of Haematology, Guy’s and St Thomas’ NHS Foundation Trust, London SE1 9RT, UK; 4 Department of Clinical and Experimental Medicine, University of Catania, 95123 Catania, Italy; 5 Department of General Surgery and Medical-Surgical Specialties, University of Catania, 95123 Catania, Italy; 6 Department of Precision Medicine in Medical, Surgical and Critical Care (Me.Pre.C.C.), University of Palermo, 90128 Palermo, Italy; 7 Department of Medical Surgical Sciences and Advanced Technologies “G.F. Ingrassia”, University of Catania, 95123 Catania, Italy; 8 Hematology and Bone Marrow Transplantation Unit, Hospital of Bolzano (SABES-Azienda Sanitaria dell’ Alto Adige), Teaching Hospital of Paracelsus Medical University, 39100 Bolzano, Italy; calogero.vetro@sabes.it

**Keywords:** acute myeloid leukemia, next-generation sequencing, prognosis, Sangel AML model, TP53

## Abstract

**Background**: Acute myeloid leukemia (AML) is genomically heterogeneous, and translating baseline molecular data into individualized prognosis remains difficult. We assessed real-world outcomes and externally validated the Sanger Institute AML multistage prognostic model. **Methods**: This single-center, retrospective study included 73 AML patients who underwent targeted NGS profiling. In intensively treated patients, the published, validated Sanger AML multistage prognostic model was compared with observed 12- and 36-month clinical outcomes using quadratic-weighted Cohen’s kappa. **Results**: Median age was 61 years, and median overall survival was 13 months, with the most significant survival differences driven by treatment intensity. *TP53* mutations occurred in 7 patients (9.6%) and were linked to primary refractoriness and extremely poor survival. *TP53* was the only independent predictor of death (HR 8.07, 95% CI 2.23–29.13; *p* = 0.0014). Model concordance was moderate at 12 months (29 evaluable cases; weighted κ = 0.52; alive/dead κ = 0.52) and fair-to-moderate at 36 months (23 cases; weighted κ = 0.46). The tool performed best for predicted death without remission, while most discrepancies involved patients expected to remain in first remission who later relapsed and died. **Conclusions**: *TP53* disruption dominates prognosis in real-world AML. The multistage tool supports early high-risk identification but shows limited long-term calibration, motivating the development of dynamic models integrating contemporary therapies and longitudinal min/serial NGS data.

## 1. Introduction

The genomic complexity of acute myeloid leukemia (AML) reflects a vast range of driver mutations, co-occurring variants, and subclonal hierarchies that influence disease behavior and therapeutic response. Traditional classification and risk-stratification systems, largely based on cytogenetic and limited molecular testing, capture only part of this complexity, leaving many patients unclassified or inaccurately risk-assigned [1,2,3,4,5].

The advent of next-generation sequencing (NGS) has profoundly transformed the diagnostic and prognostic landscape of AML [6,7,8]. NGS enables comprehensive profiling of somatic mutations, the detection of rare or co-occurring variants, and the assessment of clonal evolution, offering unprecedented insights into disease biology. The integration of NGS into clinical practice has refined AML classification and risk assessment, leading to the identification of novel molecular subgroups and improved prognostic stratification [9,10]. The 2022 European LeukemiaNet (ELN) and the International Consensus Classification now incorporate extended mutational profiling, enabling more accurate outcome prediction and better guidance for therapeutic decisions, including the selection of candidates for allogeneic hematopoietic stem cell transplantation [9,10,11,12,13].

Recent evidence strongly recommends integrating advanced molecular diagnostics, including NGS, into the initial evaluation of AML to optimize classification and risk-adapted management [9]. Beyond diagnosis and risk classification, NGS could play a pivotal role in monitoring measurable residual disease (MRD), offering greater sensitivity than traditional PCR-based or flow cytometric methods. This could enable dynamic risk assessment throughout the disease course and allow for real-time therapeutic adjustments [14,15,16].

However, even with increasingly detailed molecular characterization, translating such complex genomic information into individualized prognostic estimates remains challenging. A significant step forward in this direction was provided by Gerstung et al., who developed a multistage prognostic model for AML using a knowledge-bank approach [17]. Using data from more than 1500 patients treated with intensive chemotherapy, this model predicts patient-specific disease trajectories through multiple clinical stages, including induction death, complete remission, relapse, post-relapse survival, and death without remission, by estimating transition hazards between these states. Variables at diagnosis include genomic and karyotype evaluation, associated with clinical and demographic data (such as age, blood count, blast rate, splenomegaly, AML type, and initial clinical intention regarding whether to refer the patient for allogeneic hematopoietic stem cell transplant). This multistate framework integrates both baseline and dynamic factors, outperforming traditional risk scores such as ELN in discriminative ability (Harrell’s C-index ≈ 0.72 vs. 0.64) [17].

The model was subsequently implemented by the Sanger Institute as a publicly available web-based tool, allowing clinicians and researchers to estimate individualized probabilities of survival and remission over time based on patient-specific genomic and clinical variables. It has indeed undergone initial validation and external evaluation. In the original publication, the model was tested using leave-one-out cross-validation and external data from The Cancer Genome Atlas (TCGA, n = 176), achieving consistent concordance indices around 0.70–0.72, confirming its predictive performance beyond the original cohort [17]. Subsequent work by Huet et al. has applied the knowledge-bank predictions retrospectively in independent patient cohorts, further supporting its prognostic value in real-world AML settings [18].

There was also a reproducibility assessment that demonstrated partial replication of model outcomes and highlighted the robustness and challenges of translating the algorithm into independent computational environments [19].

However, its predictive accuracy across different timepoints remains to be clearly established. We aimed to evaluate the clinical and molecular outcomes of AML patients in a real-world, single-center cohort, integrating comprehensive NGS profiling and focusing primarily on the external validation of the Sanger AML multistage prognostic model to identify its strengths and limitations in contemporary clinical practice.

## 2. Materials and Methods

### 2.1. Aim and Study Design

This retrospective, monocentric study evaluated the clinical and molecular outcomes of patients diagnosed with AML across all disease stages and age groups. The primary objective of this study was to externally validate the Sanger AML multistage prognostic model, with additional analyses evaluating the relationship between detailed mutational profiles obtained via NGS and patient outcomes [17]. Alive patients provided written informed consent for data collection, performed in accordance with the Declaration of Helsinki.

Based on the multistage prognostic model, beyond conventional survival endpoints, the study aimed to delineate patient trajectories into clinically meaningful categories at 12 and 36 months, if evaluable, and to assess the predictive power of molecular and clinical parameters within a multistage framework.

Patients were eligible if they had a confirmed diagnosis of AML according to the WHO 2022 criteria and an available NGS myeloid panel study. Exclusion criteria included insufficient biological material for molecular profiling, incomplete clinical documentation, and a follow-up period of less than 12 months for those alive at the first timepoint. All patients underwent comprehensive baseline assessments, including bone marrow aspiration and/or biopsy, complete blood counts, biochemical panels, and cytogenetic studies.

### 2.2. DNA Isolation and Molecular Characterization

Samples of bone marrow or peripheral blood were collected from each patient in EDTA tubes and analyzed for molecular profiling. Genomic DNA was extracted from 0.7 mL of whole blood using the QIAsymphony DSP DNA Midi kit and quantified with a Qubit 3.0 fluorometer. For RNA extraction, red blood cells were removed by two consecutive lysis steps (15 min on ice) followed by centrifugation; white blood cells were then washed, resuspended in PBS, counted by CytoFLEX flow cytometry, and 1 × 10^7^ cells were lysed in RLT buffer and stored at −80 °C. Total RNA was subsequently extracted using an automated QIAsymphony protocol and quantified by Qubit.

Target enrichment and library preparation were performed with the Oncomine™ Myeloid Research Assay (Chef Ready) on the Ion AmpliSeq™ Chef platform. This panel detects mutations in 40 myeloid-relevant genes (e.g., NPM1, FLT3, DNMT3A, IDH1/2, TP53) and fusion transcripts from 29 driver genes in a single NGS run. For each sample, 10 ng of DNA or RNA (15 µL) were barcoded, and libraries were automatically prepared and pooled on the Ion Chef instrument, quantified by Qubit, and sequenced on an Ion GeneStudio S5 Plus using an Ion 530 Chip. Sequencing was performed with massively parallel sequencing of amplicons 127–299 bp in length, achieving vertical coverage of at least 500× per amplicon. Variant calling and annotation were performed using Ion Reporter Software v5.20, comparing sequences to the hg19 reference genome, excluding germline variants and low-allele-frequency mutations (<2%). Patients were stratified by mutational burden according to ELN 2017 criteria [20].

### 2.3. Clinical Treatment and Response Assessment

Treatment was administered according to patient fitness and disease risk [9]. Fit patients typically received intensive induction chemotherapy, most commonly the standard “7 + 3” regimen (cytarabine plus daunorubicin), with or without midostaurin, depending on FLT3 mutation occurrence, or the liposomal formulation Vyxeos^®^ (daunorubicin/cytarabine), designed for t-AML (AML related to prior therapy) and AML-MRC (AML with myelodysplasia-related changes) according to the 2016 WHO classification [21,22]. For older or less fit patients, hypomethylating agents, such as azacitidine or decitabine, were combined with the BCL-2 inhibitor venetoclax. Hematopoietic stem cell transplantation was performed in young high-risk patients or after salvage therapy in case of relapsed/refractory patients. Palliative approaches were considered for frail patients. Potential second-line therapies included salvage chemotherapy or combinations of hypomethylating agents with venetoclax or targeted inhibitors, such as the FLT3 inhibitor gilteritinib for patients with relevant mutations [23,24]. Treatment responses were assessed 21–28 days post-induction using the 2003 IWG criteria [25]. Complete remission (CR) was defined as the absence of leukemic blasts in the bone marrow with recovery of peripheral blood counts, while complete remission with incomplete hematologic recovery (CRi) indicated normalization of marrow blasts without full recovery of peripheral counts. Morphologic leukemia-free state (MLFS) was characterized by <5% marrow blasts without full peripheral recovery, and partial remission (PR) reflected a substantial (more than 50% or blasts comprised between 5–20%) but incomplete reduction in blast percentage.

The status at every time point was defined as death without achieving remission, death without relapse, death following relapse, survival after relapse, survival in first complete remission, and survival without complete remission.

### 2.4. Validation of the AML Multistage Prognostic Tool

For each patient, the required input variables for the Sanger AML multistage model, including age, cytogenetic risk, mutational profile, and treatment details, were collected. Predicted survival probabilities were compared with observed outcomes across the detailed clinical categories described above. Patient-specific outcomes were manually calculated using the Sanger AML multistage tool (https://cancer.sanger.ac.uk/aml-multistage, accessed on 16 March 2025), allowing for a direct comparison between the model predictions and actual clinical trajectories.

### 2.5. Statistical Analyses

Patient outcomes were evaluated comprehensively across the entire cohort to capture the full range of AML disease trajectories. Analyses considered overall survival, disease-free survival, remission status, relapse patterns, and treatment response.

Patient demographics, treatment regimens, and molecular profiles were summarized using descriptive statistics. Survival analyses, including OS, PFS, and DFS, were performed using Kaplan–Meier estimates and log-rank tests. The differences between the groups were studied using the Mann–Whitney U test or the Kruskal–Wallis test and the chi-square test or Fisher’s exact test for continuous data and categorical data, respectively. Multivariate analysis was calculated through Cox Proportional Hazards Regression.

To evaluate the concordance between the AML multistage prognostic tool and actual patient outcomes, the tool’s predicted probabilities were compared with observed clinical outcomes. For each patient treated with intensive chemotherapy, the predicted outcomes at 12 and 36 months were expressed as a percentage for each possible event. The category with the highest predicted probability (considered only if ≥50%) was considered the expected outcome, which was then compared to the patient’s actual status (alive or deceased) to assess overall concordance. Concordance was evaluated using the weighted Cohen’s kappa (quadratic weights), which measures agreement for ordinal data beyond chance.

For 12-month predicted probabilities, because the AML multistage tool provides precise probabilities only at 36 months, values were first manually extracted from Kaplan–Meier plots generated by the Sanger AML multistage tool and then confirmed using an artificial intelligence-assisted approach. The Kaplan–Meier plots were interpreted using a computational method based on ChatGPT 5.2 (https://chatgpt.com/, accessed on 16 March 2025), allowing comparison of predicted and observed outcomes at intermediate time points. MedCalc Statistical Software version 19.2.6 (MedCalc Software bv, Ostend, Belgium; https://www.medcalc.org; 2020) and Prism 9 (version 9.5.1, 528, accessed on 30 September 2023) were used for statistical analysis.

## 3. Results

### 3.1. Patient Demographics

A total of 73 patients, including 33 females and 40 males, who were newly diagnosed with AML, were included in the study. The median age at diagnosis was 61 years [19–81], with a median follow-up duration of 10 months (range 1–61).

The distribution of AML subtypes included 44 cases of de novo AML (60%), 20 cases of AML secondary to MDS (27%) with myelodysplasia-related change (MRC), 5 cases of therapy-related AML (7%), and 4 cases evolving from myeloproliferative neoplasms (6%). Performance status, as assessed by ECOG Performance Score, was 0–2 in 66 patients (90%) and 3–4 in 7 patients (10%). Complex or high-risk karyotypes were detected in 21 of 65 evaluable patients (32%). Risk classification according to ELN 2022 was favorable in 11 cases (15%), intermediate in 17 cases (23%), and adverse in 45 cases (62%).

Regarding initial treatment, 40 patients (54%) received intensive chemotherapy (e.g., “7 + 3” or a liposomal formulation), 29 patients (40%) received non-intensive approaches, including hypomethylating agents with venetoclax, and 4 patients (6%) received palliative care. HSCT was performed in 20 patients (27%) based on disease risk or relapse occurrence.

Considering the more recent ELN 2024 guidelines regarding the stratification of older AML patients, out of 29 treated with less-intensive therapies, 20 (69%) were low-, 6 (21%) were intermediate- and 3 (10%) were high-risk [10].

Table 1 summarizes detailed clinical data, including blood counts, as well as peripheral and bone marrow blast rates.

### 3.2. Molecular Findings by NGS

NGS analysis revealed a heterogeneous mutational landscape. The most frequently mutated genes were *FLT3-ITD* and *ASXL1*, present in 18 patients (24.7%), followed by *TET2* in 14 patients (19.2%) and *DNMT3A* in 13 (17.8%). Mutations in *NPM1* were identified in 15 patients (20.5%), *RUNX1* in 12 (16.4%), and *NRAS* in 12 (16.4%). Other recurrently mutated genes included *TP53* (7 patients, 9.6%), *SRSF2* (6, 8.2%), *IDH1* (6, 8.2%), and *IDH2* (6, 8.2%).

Several patients harbored co-occurring mutations, particularly among epigenetic regulators (*ASXL1, TET2, DNMT3A*) and signaling pathway genes (*FLT3, NRAS*), as detailed in Supplementaries (Table S1 and Figure S1).

### 3.3. Treatment Outcomes

**Figure 1:**
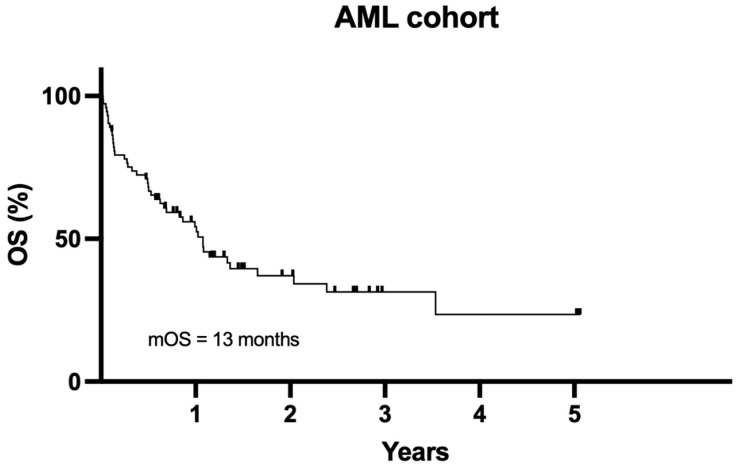
Median OS for the entire cohort.

At the 12-month follow-up, 29 (39.7%) patients were alive, and 44 (60.3%) died. The median OS for the whole cohort was 13 months (Figure 1).

At the last follow-up, evaluating differences between censored and uncensored patients, no significant difference in mOS was observed (12 months for males vs. 16 months for females, *p* = 0.824). On the other side, splitting patients according to median age, patients younger than 61 years achieved a median OS of 20 months compared with 8 months in those aged ≥61 (*p* = 0.021), as well as the performance status, assessed by ECOG score (mOS of 13 months for ECOG 0–2, compared to only 3 months in patients with ECOG 3–4, *p* = 0.02).

Focusing on disease characteristics, the AML subtype influenced prognosis: patients with de novo AML had a mOS of 16 months, whereas those with MRC- or therapy-related AML had a mOS of 7 months (*p* = 0.021). Patients without complex or high-risk karyotypes demonstrated longer survival (mOS 24 months) compared with those harboring high-risk abnormalities (mOS 12 months, *p* = 0.046).

As expected, treatment intensity had the strongest influence on outcome. Patients receiving intensive chemotherapy achieved a mOS of 20 months, whereas those treated with non-intensive regimens achieved 10 months, and those receiving palliative care achieved only 1 month (*p* < 0.001). Other variables, including ELN 2022 risk classification, did not show statistically significant differences in survival in this cohort (Table 2). Conversely, focusing only on patients treated with less intensive schemes, the ELN 2024 classification significantly divides the 3 risk classes (mOS of 12 months for low-, 8 for intermediate-, and 1 for high-risk patients, *p* < 0.001).

Regarding NGS, evaluating the single gene, only the TP53 mutation (found in 7 patients) was statistically correlated with the worst outcome (14 months vs. 1, *p* < 0.001), as detailed in Supplementary Table S1.

In multivariate Cox proportional-hazards regression, TP53 mutation emerged as a strong independent predictor of poor survival (HR 8.07, 95% CI 2.23–29.13, *p* = 0.0014), conferring more than eightfold increased risk of death. Other variables, including age, ECOG performance, AML type, and treatment intensity, were not statistically significant in the multivariate model. The overall model was highly significant (Chi-square = 29.88, df = 6, *p* < 0.0001) with good discriminative ability (Harrell’s C-index 0.732, 95% CI 0.660–0.804), indicating how *TP53* mutation is the predominant independent factor influencing outcome.

To further explore treatment outcomes, patients were stratified according to their response to initial therapy and subsequent relapse. 21 patients who achieved a complete remission (CR/CRi) subsequently relapsed, 23 patients achieved a response and remained in remission, and 29 patients never achieved a response and died or remained in active disease (Table 3).

### 3.4. Sanger Multistage Tool in Patients Treated with Intensive Approaches

At the 12-month time point, overall, 29 patients with a predicted probability ≥50% (5 deaths after relapse, 9 deaths without remission, 15 alive in first CR) were evaluable for concordance analysis between predicted and observed outcomes. The weighted Cohen’s kappa coefficient was 0.52 (SE = 0.14; 95% CI = 0.24–0.80). The highest concordance was observed among patients predicted to be alive in CR1 and those effectively remaining in continuous remission (13 cases), while most discrepancies occurred between those expected to be alive in the first CR but who experienced death after relapse (4 cases). Conversely, among patients predicted to die without remission, the majority (5 cases) actually did, confirming substantial consistency in this high-risk subgroup.

At this time point, the alive vs. dead classification showed a Cohen’s kappa of 0.52 (SE = 0.15; 95% CI 0.20–0.82), indicating moderate agreement between predicted and observed outcomes.

At the central time point of the Sanger model (36 months), 23 patients were assessable for concordance between predicted and observed outcomes (9 are alive and in ongoing follow-up). The weighted Cohen’s kappa coefficient was 0.46 (SE = 0.15; 95% CI = 0.16–0.77). The highest concordance was again observed in the subgroup predicted to experience death without remission, with 5 of these patients effectively dying without achieving remission, and in those who are in first CR (6 cases). As predicted, 4 patients experienced a relapse, to which is added one who should have been on the first CR.

A Table summarizing the results is reported in the Supplemental Files.

## 4. Discussion

This study provides an integrated clinical and molecular analysis of AML patients profiled by NGS, with a primary focus on the external validation of the Sanger AML multistage prognostic model in a real-world, monocentric Italian cohort. While AML is characterized by profound biological and clinical heterogeneity, including coexisting genomic lesions, cytogenetic abnormalities, and clonal hierarchies driving variable disease trajectories, our study demonstrates the applicability and limitations of the multistage tool in contemporary clinical practice. In parallel, we observed that only a limited subset of genetic events, most notably TP53 mutations, retained a strong independent prognostic impact across therapeutic settings, confirming previous reports. Consistent with extensive prior literature, TP53 mutation emerged in our cohort as the single most powerful predictor of adverse outcome, associated with extremely short survival and high rates of primary resistance [5,26,27]. Median overall survival in TP53-mutated cases rarely exceeds 6–9 months, even with intensive therapy or venetoclax-based combinations [28,29,30]. In our multivariate analysis, TP53 mutation conferred an eightfold higher risk of death, clearly outweighing traditional prognostic variables such as age, performance status, or treatment intensity. Novel strategies, such as p53-reactivating agents (eprenetapopt), are under investigation and may represent the most rational direction for this population [31,32,32,33,34,35,36,37].

Focusing on the validation of the Sanger AML multistage model developed by Gerstung et al. [17], in our real-world cohort, the Sanger AML multistage model demonstrated moderate concordance at 12 months (κ = 0.52) and fair concordance at 36 months (κ = 0.46). While the 36-month predicted probabilities represent the primary and officially validated outputs and form the central focus of our validation, the 12-month analysis was included as a complementary assessment to provide additional insights into early disease trajectories, such as induction failure and early relapse.

Performance was highly variable across clinical subgroups. The model performed best in patients predicted to die without achieving remission, a category driven by baseline genomic and cytogenetic features that the model effectively captures. Conversely, predictions were less accurate in patients expected to remain alive in CR1 or to experience death after relapse, where a significant number were misclassified.

Several factors can explain these discrepancies. First, the model relies exclusively on baseline variables, assuming a static risk landscape, while AML evolves dynamically through therapy and clonal selection. Additionally, among the 40 patients with intermediate/high-risk AML, 15 (37.5%) underwent HSCT and were analyzed for Sanger analysis. The low HSCT rate could partially explain the low concordance rate. Additionally, the salvage therapy after chemotherapy failure was mostly based on venetoclax-based regimens, avoiding the high-dose chemotherapy salvage. Additionally, MRD would have influenced the long-term outcome of these patients, but its analysis was not included in our study.

Based on our experience, in a real-life setting, the model appears most suitable as an early prognostic tool to identify patients at high risk of induction failure or early death, where baseline genomic and cytogenetic profiles dominate. It could be applied at diagnosis to support decisions on treatment intensity, trial referral, or early transplantation planning. In contrast, its use for long-term outcome prediction should be approached with caution, where disease trajectories are influenced by dynamic therapeutic and biological variables beyond the model’s original scope. Retraining the model on contemporary datasets that include venetoclax- and IDH/FLT3-targeted therapies, and integrating MRD and serial molecular data, could enhance its calibration and extend its applicability in current practice.

Despite these insights, our study has several limitations. The sample size was relatively modest, which restricts the power of subgroup analyses and increases uncertainty around concordance estimates. The cohort was also highly heterogeneous in terms of age, disease subtype, and therapeutic strategy, reflecting the real-world complexity of AML but limiting statistical uniformity. Finally, the multistage model does not account for time-dependent molecular dynamics such as emerging subclones or persistent mutations, which are increasingly recognized as key determinants of relapse and survival [38].

Even acknowledging these limitations, this study represents one of the few real-world external validations of the Sanger AML multistage model using comprehensive molecular data [18,39]. Our results emphasize that while static baseline predictors remain valuable for early stratification, they lose accuracy over time as the therapeutic environment and disease biology evolve. Predictive frameworks in AML should therefore move toward dynamic, longitudinal models that integrate serial NGS data, MRD kinetics, and treatment-related variables to continuously update patient-specific risk.

## 5. Conclusions

In conclusion, while TP53 mutation remains an important biological predictor of adverse outcome, this study demonstrates that the Sanger multistage model, while valuable, provides partial predictive accuracy when applied to heterogeneous, contemporary cohorts. Together, these observations call for a transition from static, baseline-only risk models toward dynamic, mutation-informed, and therapy-adaptive prognostic frameworks. Such evolution will be crucial to bridge the gap between precision genomics and personalized therapeutic decision-making in AML.

## Figures and Tables

**Table 1:** Detailed clinical data of enrolled patients.

	AML Patients (n = 73) [Range]—(%)
Sex, male/female	40/33 (55/45)
Age at diagnosis, years	61 [19–81]
Type of AML:	
De novo	44 (60)
MRC- or t-AML	29 (40)
Blood count:	
Hb (g/dL)	8.3 [3.3–15.1]
PLT (10^3^/mmc)	52 [1–352]
WBC (/mmc)	7230 [160–322,580]
Blast rate in PB (%)	35 [10–90]
Blast rate in BM (%)	55 [5–95]
ECOG PS	
0–2	66 (90)
3–4	7 (10)
Complex/high-risk karyotype:	Available for 65
Yes	21 (32)
No	44 (68)
Risk status according to ELN 2022:	
Favorable	11 (15)
Intermediate	17 (23)
Adverse	45 (62)
AML approach at diagnosis:	
Intensive chemotherapy	40 (54)
Non-intensive treatment	29 (40)
Palliative care	4 (6)
HSCT	
Yes	20 (27)
No	53 (73)
Median follow-up, months	10 [1–61]

AML: acute myeloid leukemia; MRC: myelodysplasia-related changes; t-AML: therapy-related AML; Hb: hemoglobin; PLT: platelets; WBC: white blood cells; PB: peripheral blood; BM: bone marrow; HSCT: hematopoietic stem cells transplant.

**Table 2:** Comparison between variables at baseline and OS.

	Variable	mOS (Months)	*p*
Sex	Male	12	0.824
	Female	16	
Age at diagnosis, years	<61	20	0.021
	≥61	8	
Type of AML	De novo	16	0.021
	MRC/therapy-related	7	
ECOG PS	0–2	13	0.028
	3–4	3	
Complex/high-risk karyotype	No	24	0.046
	Yes	12	
Risk status according to ELN 2022	Favorable	8	0.938
	Intermediate	NR	
	Advanced	13	
AML approach at diagnosis	Intensive chemotherapy	20	<0.001
	Non-intensive treatment	10	
	Palliative	1	
Gene mutations	Reported in Supplementary Table S1		

MRC: myelodysplasia-related changes; PS: performance score; AML: acute myeloid leukemia.

**Table 3:** Stratification of patients according to their response to initial therapy and relapse.

	CR1 Patients (n = 23) [Range]—(%)	Relapsed Patients (n = 21) [Range]—(%)	Refractory Patients (n =29) [Range]—(%)	*p*
Sex, male/female	11/12 (48/52)	14/7 (67/33)	15/14 (52/48)	0.416
Age at diagnosis, years	51 [19–74]	64 [24–77]	63 [31–81]	0.003
Type of AML:				0.2
De novo	16 (70)	15 (71)	13 (45)	
MRC/therapy-related	7 (30)	6 (29)	16 (55)	
ECOG PS				0.148
0–2	22 (96)	20 (95)	24 (83)	
3–4	1 (4)	1 (5)	5 (17)	
Complex/high-risk karyotype	Available for 22	Available for 20	Available for 23	0.07
No	19 (86)	12 (60)	13 (57)	
Yes	3 (14)	8 (40)	10 (43)	
Risk status according to ELN 2022:				0.18
Favorable	3 (13)	6 (29)	6 (21)	
Intermediate	9 (39)	3 (14)	2 (7)	
Adverse	17 (48)	12 (57)	21 (72)	
AML approach at diagnosis:				0.007
Intensive chemotherapy	18 (78)	8 (38)	14 (48)	
Non-intensive treatment	5 (22)	13 (62)	11 (52)	
Palliative care	0 (0)	0 (0)	4 (0)	
HSCT in CR1				<0.001
No	8 (35)	17 (81)	29 (100)	
Yes	15 (65)	4 (19)	0 (0)	
Median follow-up, months	18 [1–61]	4 [13–61]	2 [1–16]	
Gene mutations with significance				
*FLT3-ITD*	10 (43)	4 (19)	4 (14)	0.037
*TP53*	0 (0)	0 (0)	7 (100)	0.003

MRC: myelodysplasia-related changes; PS: performance score; AML: acute myeloid leukemia; HSCT: hematopoietic stem cell transplant; CR1: first complete remission.

## Data Availability

The original contributions presented in the study are included in the article. Further inquiries can be directed to the corresponding author.

## Supplementary Materials

The following supporting information can be downloaded at: https://www.mdpi.com/article/10.3390/genes17020218/s1.
