# Supplementary material for: Integrating Genomic and Clinical Data in AML: Real-World Application of the Sanger Multistage Model
Source: Genes (Basel). 2026 Feb 10;17(2):218. doi: 10.3390/genes17020218 (PMC12941301; doi:10.3390/genes17020218)
Supplement: Supplementary file 1 [file genes-17-00218-s001.zip › genes-4071096-supplementary.pdf]

Supplementary Table S1.

Gene		mOS (months) [patients]	p
NPM1	WT	12 [61]	0.3
	Mutated	20 [12]	
FLT3-ITD	WT	12 [55]	0.048
	Mutated	NR [18]	
ASXL1	WT	12 [55]	0.578
	Mutated	14 [18]	
TET2	WT	12 [59]	0.552
	Mutated	13 [14]	
DNMT3A	WT	12 [60]	0.262
	Mutated	20 [13]	
RUNX1	WT	13 [61]	0.667
	Mutated	13 [12]	
NRAS	WT	13 [61]	0.388
	Mutated	7 [12]	
TP53	WT	14 [66]	<0.001
	Mutated	1 [7]	
SRSF2	WT	13 [67]	0.683
	Mutated	8 [6]	
IDH1	WT	13 [67]	0.952
	Mutated	8 [6]	
IDH2	WT	13 [67]	0.803
	Mutated	12 [6]	
KRAS	WT	13 [68]	0.111
	Mutated	6 [5]	

Supplementary Figure S1.

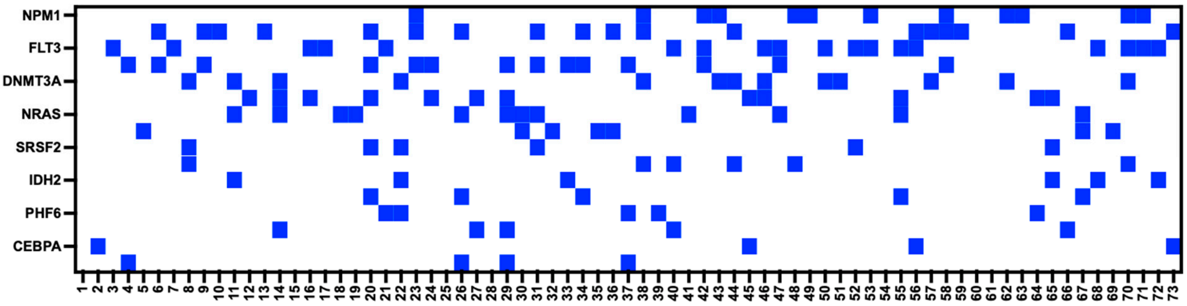

Supplementary Table S2.

Timepoint	Predicted Outcome (≥50%)	Number of Patients	Observed Alive	Observed Dead	Notes / Concordance
12 months	Alive in first CR	15	13	2	High concordance for continuous remission
12 months	Death after relapse	5	0	5	Fully concordant in high-risk subgroup
12 months	Death without remission	9	0	9	Fully concordant in high-risk subgroup
36 months	Alive in first CR	6	6	0	Concordant
36 months	Death after relapse	5	1	4	Partial discordance: one patient misclassified
36 months	Death without remission	5	0	5	Fully concordant
36 months	Alive, ongoing follow-up	7	7	0	Not yet events, counted separately
